# Immune-Boosting, Antioxidant and Anti-inflammatory Food Supplements Targeting Pathogenesis of COVID-19

**DOI:** 10.3389/fimmu.2020.570122

**Published:** 2020-10-07

**Authors:** M. Mrityunjaya, V. Pavithra, R. Neelam, P. Janhavi, P. M. Halami, P. V. Ravindra

**Affiliations:** ^1^Department of Biochemistry, Council of Scientific and Industrial Research-Central Food Technological Research Institute, Mysuru, India; ^2^Academy of Scientific and Innovative Research, Ghaziabad, India; ^3^Department of Microbiology and Fermentation Technology, Council of Scientific and Industrial Research-Central Food Technological Research Institute, Mysuru, India

**Keywords:** SARS-CoV-2, COVID-19, pathogenesis, food supplements, immune-boosting, antioxidant, anti-inflammation

## Abstract

The COVID-19 is an acute and contagious disease characterized by pneumonia and ARDS. The disease is caused by SARS-CoV-2, which belongs to the family of *Coronaviridae* along with MERS-CoV and SARS-CoV-1. The virus has the positive-sense RNA as its genome encoding for ~26 proteins that work together for the virus survival, replication, and spread in the host. The virus gets transmitted through the contact of aerosol droplets from infected persons. The pathogenesis of COVID-19 is highly complex and involves suppression of host antiviral and innate immune response, induction of oxidative stress followed by hyper inflammation described as the “cytokine storm,” causing the acute lung injury, tissue fibrosis, and pneumonia. Currently, several vaccines and drugs are being evaluated for their efficacy, safety, and for determination of doses for COVID-19 and this requires considerable time for their validation. Therefore, exploring the repurposing of natural compounds may provide alternatives against COVID-19. Several nutraceuticals have a proven ability of immune-boosting, antiviral, antioxidant, anti-inflammatory effects. These include Zn, vitamin D, vitamin C, curcumin, cinnamaldehyde, probiotics, selenium, lactoferrin, quercetin, etc. Grouping some of these phytonutrients in the right combination in the form of a food supplement may help to boost the immune system, prevent virus spread, preclude the disease progression to severe stage, and further suppress the hyper inflammation providing both prophylactic and therapeutic support against COVID-19.

## Severe Acute Respiratory Syndrome-Coronavirus (SARS-CoV)-2 Infection

The coronavirus disease (COVID-19) was first reported in late 2019 from Wuhan's city in China. Thus far, the infection has spread to almost all countries globally and was declared a pandemic by the WHO. While writing this review, there were more than 23M confirmed cases and more than 800K deaths. In India, there were more than 3M positive cases, and more than 57K reported deaths. The mortality rate of 2–16%, the rapid spread of the disease and high mortality in the susceptible population (mainly aged over 60 years and also in patients with underlying medical conditions including diabetes, cardiovascular diseases, etc.,) has resulted in a global lockdown and life has come to a standstill causing yet another world economic recession after 2008. The incubation period is presumed to vary between 2 and 14 days. The transmission mode includes surface contact of aerosol droplets from infected persons, followed by touching nose, eyes, and mouth. Evidence also points toward vertical transmission to new-borns, also by fecal transmission (1–3). Coronaviruses are enveloped and possess positive-sense single-stranded RNA (+ssRNA) as their genome. These viruses belong to the large family of *Coronaviridae* and subfamily *Coronavirinae*, which infect birds and mammals. The genome size of these viruses ranges from 26 to 32 kb (4). The virus binds to angiotensin-converting enzyme 2 (ACE2) receptors on cells through its spike (S) glycoprotein. The S protein has two domains S1 and S2. S1 binds to the peptidase domain of ACE2, which is called the receptor-binding domain (RBD), while S2 catalyzes the membrane fusion, thereby releasing the genetic material into cells (5). Inside the cell, the RNA provides the template for structural proteins such as replicase (R1a/ab), envelope (E), Spike (S), membrane (M), nucleoprotein (N), and several non-structural proteins (NSPs 1–16), uncharacterized protein 14, protein 9b (6). Of them, non-structural proteins are predicted to participate in the host-protein interactions and modulate host cell signaling pathways. The onset of clinical disease and its progression to the severe stage may vary between individuals and that depend upon their immune status, and the presence of underlying medical conditions. In general, the typical clinical symptoms include, dry cough (67%), fever (88%), fatigue (38%), myalgias (14.9%), Dyspnoea (18.7%), other symptoms include, headache, sore throat, rhinorrhoea, and gastrointestinal symptoms. Pneumonia is severe manifestation of the infection (2).

## Pathogenesis of COVID-19

The details of the pathogenesis of SARS-CoV-2 infection is not clearly understood. The available evidence suggests that the pathogenesis of infection can be classified into two phases. Phase 1: An asymptomatic phase with or without detectable virus. Phase 2: Symptomatic phase with high viral load (4). The virus enters the airway epithelium after binding its S protein to the ACE2 receptors and subsequent priming by the cellular transmembrane protease, serine 2 (TMPRSS2). Following its entry, the virus inhibits or delays the host innate interferon (IFN) immune response. The mechanisms of how it modulates the host IFN response is not completely understood. Available evidence from other members of the same family suggests that, the virus inhibits the production of type 1 IFN as well as the signaling downstream of the interferon-α/β receptor (IFNAR) (7). The virus interferes with downstream signaling by ubiquitination and degradation of RNA sensor adaptor molecules such as mitochondrial antiviral-signaling (MAVS) protein and tumor necrosis factor receptor-associated factors (TRAF) 3/6 and inhibiting interferon regulatory factor (IRF) 3 nuclear translocation (8). Once type 1 IFN is secreted, the virus interferes IFN signaling by inhibiting signal transducer and activator of transcription (STAT) 1 phosphorylation (9). The viral proteins that modulate host type 1 IFN responses include structural (such as M, N) and NSPs. Following the impairment in the IFN system, virus replication ensues in cells. The viral replication, in turn, triggers the activation of monocytes, macrophages, granulocytes resulting in the hyper inflammatory condition described as “cytokine storm” with the massive secretion of pro-inflammatory cytokines including interleukin (IL)-1, IL-6, IL-8, IL-12, tumor necrosis factor (TNF)-α, etc. This results in hyper inflammation of tissues and subsequent tissue fibrosis and pneumonia (4, 7, 10). Studies also indicate the involvement of oxidative stress in the pathogenesis of COVID-19. Available evidence suggests that, SARS-CoV-2 infection causes oxidative stress directly by enhancing the production of reactive oxygen species (ROS) (11) and indirectly by suppressing the host antioxidant defense mediated by the nuclear factor (erythroid-derived 2)-like 2 (NRF-2) (10). Further, granulocytosis in response to SARS-CoV-2 infection also contributes to the production of super oxide ions, a type of ROS and for the additional production of pro-inflammatory cytokines (12). In a study by Lin et al. (13) showed that a viral protease 3CLpro causes a significant increase in the ROS production in HL-CZ cells. Further, study also found that elevated oxidative stress results in activation apoptosis and inflammation. In another study done on human HCoV-229E infection shows that deficiency in the expression of NRF-2 target, glucose-6-phosphate dehydrogenase (G6PDH) results in enhanced ROS as well as virus production (14). Incidentally, the NRF-2 levels were found to be suppressed in lung biopsies from COVID-19 subjects, on the other hand NRF-2 activators found to inhibit replication of SARS-CoV-2 and the inflammatory response (10). However, it is not known how SARS-CoV-2 infection causes suppression of NRF-2 signaling. Additionally, studies also suggest that SARS-CoV-2 infection triggers the activation of NF-κB-toll-like receptor (TLR) signaling pathways to induce the oxidative stress and hyper inflammatory response, ultimately leading to acute lung injury (11).

**Figure 1:**
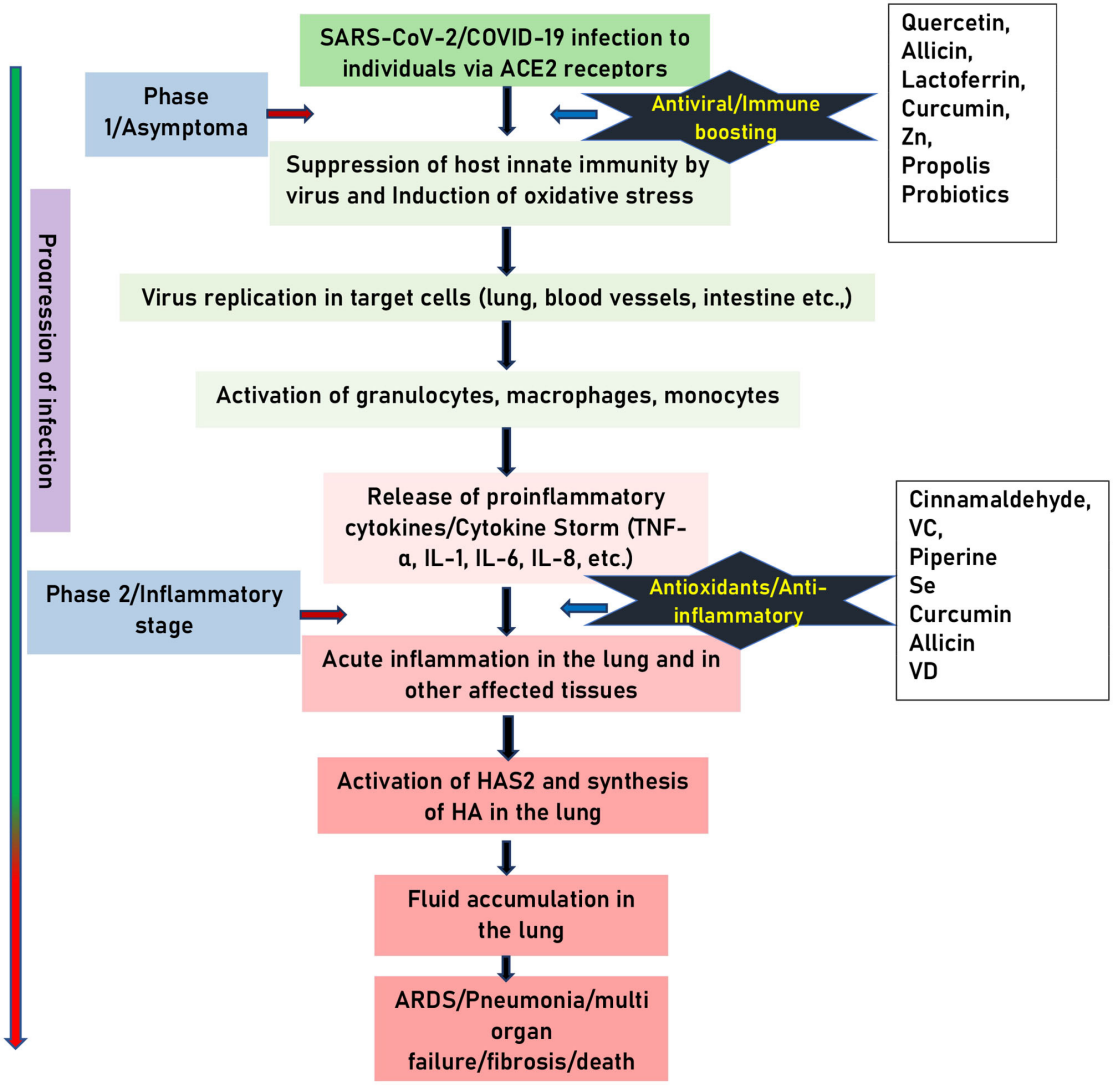
Schematic representation of pathogenesis of COVID-19. SARS-CoV-2 infection involves two phases: (1) Asymptomatic carrier phase. (2) Symptomatic inflammatory phase. The black stars indicate the stage at which food supplements can counteract the pathogenesis of COVID-19. Arrow on the left indicate the progress of the infection.

The elevated cytokines also trigger induction of endothelium HA-synthase-2 (HAS2) in alveolar epithelial cells (type 2), and fibroblasts (15). Most importantly, key molecule hyaluronan (HA) has high water binding capacity up to 1,000 times its molecular weight. Perhaps the accumulation of fluid in the lungs could be the reason that computer tomography (CT) images of the lung in acute respiratory distress syndrome (ARDS) patients show the presence of distinguishing white patches called ground glass (16). The majority of autopsies have shown that infected lungs are filled with clear liquid jelly, which resembles the lungs of wet drowning (17). Even though the nature of clear liquid jelly is not yet been determined, HA is associated with ARDS (18). The lungs of COVID-19 patients show elevated levels of inflammatory cytokines (IL-1, TNF-α). This correlates with increased activity of HAS2 and the subsequent lung pathology induced by the SARS-CoV-2 infection. Therefore, the above clinical and research findings suggest that COVID-19 pathogenesis involves two phases: Phase 1, suppression of innate immune response, increases in oxidative stress and phase 2 acute inflammation-driven damaging phase (Figure 1).

## Strategies to Counteract the SARS-CoV-2 Infection Using Food Supplements

From the point of prevention, phase 1 is crucial as individuals in this stage are carriers, they can spread the infection unknowingly. Management of individuals in phase 1, along with mounting specific adaptive immune response, and use of antivirals is critical to prevent the virus entry, replication as well as the disease progression to phase 2. Therefore, global strategies may include administration of external antiviral, and or immune-boosting food supplements. During the phase 2 of the infection, in addition to maintaining the general health condition of affected patients, the line of treatment may be focused on adapting the strategies including the use of nutritional supplements that can suppress the ongoing oxidative stress, acute-inflammation and cytokine storm so that destruction and damage caused to affected tissues is prevented. In summary, in addition to symptomatic treatment, strategies to counteract the SARS-CoV-2 infection is to boost the immune response in phase 1 while suppressing it in the second phase could be effective.

## Immune-Boosting, Antioxidant and Anti-Inflammatory Food Supplements Against COVID-19

Currently, there is one vaccine; Sputnik V, approved by the Ministry of Health, Russian Federation. It was fast-tracked for use as a corona vaccine, but experts have expressed concern about the vaccine's efficacy and safety since it has not yet been evaluated in phase 3 clinical trials. Currently, most countries around the world are into developing corona vaccines, a few of them have entered into human trials while most of them are in various stages of research and development. Further, there no specific drug for use against COVID-19 as well as substantial data both at the national or international level on the effects of nutritional supplements on risk or severity of COVID-19. The development of new antivirals for COVID-19 is a great challenge and needs a considerable length of time and effort for designing and validation. Several shreds of evidence indicate that many nutritional supplements from various spices, herbs, fruits, roots, and vegetables can reduce the risk or severity of a wide range of viral infections by boosting the immune response, particularly among people with inadequate dietary sources and also by their anti-inflammatory, free radical scavenging, and viricidal functions. These nutrients can be repurposed in mitigating the pathological effects induced by the SARS-CoV-2 infection. Therefore, the use of natural compounds may provide alternative prophylactic and therapeutic support along with the therapy for COVID-19. In the following section, the beneficial effects of some of the nutrients are described.

## Zinc (Zn)

Zinc is an essential metal involved in a variety of biological processes due to its function as a cofactor, signaling molecule, and a structural element. It regulates inflammatory activity and has antiviral and antioxidant functions (19). Studies in the rat model show that deficiency of Zn increases oxidative stress, pro-inflammatory TNF-α and vascular cell adhesion molecule (VCAM)-1 expression and causes lung tissue remodeling which was partially reversed by the Zn supplementation (20). Zn deficiency shows up-regulation of TNF-α, IFN-γ, and FasR signaling and induction of apoptosis in lung epithelial cells (21) and also up-regulates the Janus kinase (JAK)-STAT signaling in lungs under septic conditions (22). Zinc can also modulate the viral entry, fusion, replication, viral protein translation and virus budding of respiratory viruses (19, 23). Speth et al. (24) demonstrated that Zn exposure (100 μM) was shown to reduce recombinant human ACE-2 activity in rat lungs. Zn^2+^ cations especially in combination with Zn ionophore pyrithione were shown to inhibit SARS-coronavirus RNA polymerase (RNA dependent RNA polymerase, RdRp) activity by suppressing its replication (25). Studies have shown that oral supplementation of Zn reduces the occurrence of acute respiratory infections by 35%. Zn also shortens the duration of flu-like symptoms by 2 days as well as improves the rate of recovery (26). The recommended dose from various studies ranges from 20 to 92 mg/week (27). Zinc is considered as the potential supportive treatment against COVID-19 infection due to its anti-inflammatory, antioxidant as well as direct antiviral effects (28).

## Vitamin D (VD)

VD a fat-soluble vitamin, plays a vital role in both in immunomodulatory, antioxidant and antiviral responses (29, 30). The human airway epithelium constitutively expresses the vitamin D receptor thereby enabling the protective effects of VD against respiratory infections. VD blocks NF-κB p65 activation *via* up-regulation of NF-κB inhibitory protein I-kappa-B-alpha (I*K*B-α) (31). VD also decreases the expression levels of pro-inflammatory type 1 cytokines such as IL-12, IL-16, IL-8, TNF-α, IFN-γ while increasing type 2 cytokines such as IL-4, IL-5, IL-10, and regulatory T cells (32, 33). VD increases the levels of antioxidant NRF-2 and facilitates balanced mitochondrial functions, prevents oxidative stress-related protein oxidation, lipid peroxidation and DNA damage (30).

Epidemiological data relates VD deficiency to increases in the susceptibility to acute viral respiratory infections (34) while its supplementation potentiates the innate immune responses to respiratory viral infections including those caused by Influenza A and B, parainfluenza 1 and 2, respiratory syncytial virus (RSV), and chronic hepatitis C (35, 36). Though there are no reports that VD directly affects the virus replication or viral load, studies reveal that VD could contribute to antiviral activity through suppression of virus-induced inflammation. Perhaps this function of VD could help in suppression of the cytokine storm in SARS-CoV-2 infection. In a randomized controlled trial (RCT), VD supplementation of monthly high-dose (100,000 IU/month) in comparison to a standard dose (12,000 IU/month) helps in reducing the incidence of acute respiratory infections especially in older long-term care residents (37). Furthermore, evidence also suggests that VD can supplement the effectiveness of drug treatment as observed in the case of ribavirin therapy for treatment-naïve patients with chronic Hepatitis C virus (HCV) genotype 1 and HCV genotype 2e3 infections (33, 34, 38, 39). The beneficial effect of supplementation was seen in patients across all ages groups and in individuals with pre-existing chronic illness (40). Older people are most often deficient in these important micronutrients. Thus they can derive the most significant benefit from the VD supplementation (41).

## Vitamin C (VC)

Vitamin C can potentially protect against infection due to its essential role on immune health (42). This vitamin supports the function of various immune cells and enhances their ability to protect against infection. Supplementing with VC has been shown to reduce the duration and severity of upper respiratory infections (most of which are assumed to be due to viral infections), including the common cold (43). The recommended dose of VC varied from 1 to 3 g/day. The total recommended daily allowance (RDA) for VC is 60 mg. Various spices, herbs, fruits, and vegetables have found to be excellent sources of VC (44). For example, thyme fresh (267%RDA), turmeric (43%RDA), cardamom (35%RDA), coriander (35%RDA), beetroot juice are good sources of VC (45). VC is also a potent antioxidant. As an antioxidant, it scavenges ROS, prevents lipid peroxidation, and protein alkylation and thus protects cells from oxidative stress induced cellular damage (46). Studies also have revealed that administration of VC in combination with quercetin provides synergistic antiviral, antioxidant and immunomodulatory effects (47). Recently, based on the clinical trial it is proposed that the oral administration of 250–500 mg quercetin, 500 mg VC for high risk and mild symptomatic subjects twice a day for 7 days and up to 3 g VC and 500 mg quercetin twice a day for 7 days in ARDS patients (assisted ventilation/intubation) improves the overall recovery in SARS-CoV-2 subjects (47). Therefore, having the food supplement incorporated with sources of VC can help in alleviating and providing immune boosting as well as an anti-inflammatory, antioxidant effect against SARS-CoV-2 infection (48).

## Curcumin

Curcumin has a broad spectrum of biological actions, including antibacterial, antiviral, antifungal, antioxidant and anti-inflammatory activities (49). It inhibits the production of pro-inflammatory cytokines (IL-6 and TNF- α) in lipopolysaccharide (LPS)-stimulated BV2 microglial cells (50) and IL- 1β and IL-6 in TNF-α treated HaCaT cells *via* inhibiting the NF-κB and MAPK signaling pathways (51). The curcumin also inhibits cyclooxygenase-2 (COX-2), as well as STAT signaling pathways (52). Curcumin exerts antiviral effect on a broad range of viruses including influenza virus, adenovirus, hepatitis, human papilloma virus (HPV), human immunodeficiency virus (HIV), herpes simplex virus−2 (HSV-2) and Zika viruses (53). It exerts antiviral effect by various mechanisms ranging from inhibiting the virus entry into cells, inhibiting encapsulation of the virus and viral protease, inhibiting the virus replication, as well as modulating several signaling pathways (54). Recent study has shown that curcumin potentially inhibits ACE2, modulates characteristics of lipid bilayer, as well as viral S protein inhibiting entry of virus into cells (54, 55), inhibits the viral protease (56), stimulates host interferon production to activate the host innate immunity (55), etc. Furthermore, curcumin is a potent antioxidant. It exerts its antioxidant effects both by neutralizing free radicals and enhancing the production of antioxidant enzymes (57–60). These studies reveal potential immune-boosting, antioxidant and anti-SARS-CoV-2 effects of curcumin. Therefore, curcumin could be a potential supplement in combating the COVID-19 pathogenesis.

## Cinnamaldehyde

Cinnamaldehyde is a naturally present organic compound abundantly found in essential oils in cinnamon. It predominantly exists in the trans-isomer form, which gives cinnamon its flavor and odor (61). Cinnamaldehyde is a well-known dietary phytonutrient, known to possess anti-inflammatory properties. In a study by Liao et al. (62), it was found that cinnamaldehyde inhibits the TNF-α-induced inflammation through suppression of NF-κB activation. Studies have also found that it can suppress endotoxin-mediated hyperexpression of TLR4 and NOD-, LRR- and pyrin domain-containing protein 3 (NLRP3) inflammasome signaling pathways (63). Cinnamaldehyde is also known to downregulate the production of prostaglandins (PGEs) by downregulating IL-1β-induced COX-2 activity thus lowering the chances of hyper inflammation in a dose-dependent manner (64). All the above evidences show cases that cinnamaldehyde is a potential anti-inflammatory bioactive compound and could be useful in mitigation of SARS-CoV-2 induced hyper inflammation in the lung.

## Allicin

Garlic is a well-known plant/ herb classified under Allium (onion) family and has been used from ages for its several nutraceutical properties. The predominant thiosulfinate in fresh garlic extract identified as allicin, has shown a number of health benefits due to its anti-inflammatory, antioxidant and antiviral properties. Allicin suppresses the inflammation *via* inhibiting the TNF-α induced expression levels of IL-1β, IL-8, IP-10, and IFN-γ and also through suppression of degradation of NF-κB inhibitory protein IκB in intestinal epithelial cells (65). It inhibits inducible NO nitric oxide synthase expression in activated macrophages (66, 67). Several garlic associated compounds have found to possess a strong viricidal activity against a wide range of viruses including parainfluenza virus type 3, human rhinovirus, herpes simplex virus (HSV)-1, HSV-2, and vesicular stomatitis virus (VSV). Some of the garlic compounds that show viricidal activity are ajoene, allicin, allyl, methyl thiosulfinate and methyl allyl thiosulfinate (68, 69). Most of the above-mentioned functional effects were observed at 200 ng/ml concentrations. Studies also have found that only fresh samples with no processing such as heat induction or drying were successful to induce most of the biological activities of garlic (70). Therefore, fresh garlic extract may be useful as a prophylactic against COVID-19.

## Piperine

Black pepper has long been used in many cuisines and it holds a very valuable space among medicinal plants. Piperine that is obtained from ethanolic extract of black pepper and is a major alkaloid in the group of cinnamamides (71). Piperine possesses a strong anti-inflammatory function and therefore can be repurposed for suppression of hyper inflammation induced during COVID-19. It downregulates PGEs by inhibiting the expression levels of IL-6 and matrix metalloproteinases (MMP-13) (71). Piperine promotes innate immunity by promoting the phagocytic activity of phagocytes and is known to inhibit LPS-induced expression of IRF-1 and IRF-7 mRNA, phosphorylation of IRF-3, type 1IFN mRNA, and down-regulation of STAT-1 activity (72). Few studies conducted on microglial cells have shown that piperine inhibits LPS-Induced TNF-α, IL-6, IL-1β, and PGE2 production in BV2 cells (73). Also, it found to inhibit the production of IL-2, and IFN-γ in human peripheral blood mononuclear cells (PBMCs) (74). Furthermore, piperine treatment found to reduce the production of pro-inflammatory cytokines such as IL-1β, IL-6, TNF-α, COX-2, nitric oxide synthase-2, and NF-κB in the cerebral ischemia-reperfusion-induced inflammation rat model (75). These findings indicate the strong anti-inflammatory activity of the piperine. Further, piperine is a potent antioxidant and protects against oxidative damage by neutralizing free radicals, ROS, and hydroxyl radicals. It scavenges superoxide radicals with IC_50_ of 1.82 mM and inhibits lipid peroxidation with IC_50_ of 1.23 mM. These results indicate that piperine possesses a direct antioxidant effect against various free radicals (76). Because of these properties, piperine can be tried as a prophylactic or therapeutic compound to protect from the oxidative stress and hyper inflammation induced during the COVID-19.

## Selenium (Se)

Selenium is abundantly found in common foods such as corn, garlic, onion, cabbage, broccoli. It's an essential micronutrient that plays a vital role in various physiological processes and on the immune system. Selenium exerts its biological effect through incorporation into selenoproteins in the body. Optimum selenium status (100 μg per day) promotes enhanced T cell proliferation, NK cell activity and innate cell functions. Further supports stronger vaccine response and robust immunity to pathogens. Also, suppresses severe inflammation in tissues such as lungs and intestine (77). Studies have shown that selenium supplementation modulates the inflammatory response in respiratory distress syndrome patients by restoring the antioxidant status of the lungs and suppressing the IL-1β and IL-6 levels (78). Selenium supplementation suppresses pathogen induced activation of NF-κB and its downstream pro-inflammatory cytokine release (79). The antiviral properties of selenium have found to be mediated through its antioxidant effects. Selenium-deficient HIV+ patients tend to present with diminished antioxidant glutathione peroxidase activity (77). On the other hand, selenium supplementation demonstrates the improved CD+ T cell counts (80) and improves glutathione peroxidase and other antioxidant selenoenzymes along with catalase activities (81). Overall, selenium improves the immunity through its non-enzymatic role acting as cofactor for enzymes involved in critical post-translational modifications of proteins. Because of its substantial role in suppressing the inflammation and augmentation of antioxidant status and innate immunity, selenium supplementation may be useful in fight against COVID-19.

## Propolis

Propolis produced by honeybees and known to have a broad spectrum of biological properties, including anti-microbial, anti-inflammatory, dermatoprotective, laxative, anti-diabetic, anti-tumor, and immunomodulatory activity (82). The immunomodulatory activity is attributed to flavonoids and some phenolic acids mainly caffeic acid phenethyl esters and artepillin C (3,5-diprenyl-4-hydroxycinnamic acid). Propolis exhibits immunomodulatory effects on a broad spectrum of immune cells mediated by the modulation of extracellular signal-regulated kinase 2 and MAPK signaling pathways. Further, it also modulates nuclear factor of activated T cells (NFAT) and NF κB signaling pathways (82, 83). Propolis also stimulates greater antibody production, suggesting that it could be used as an adjuvant in vaccines. Propolis at higher concentration inhibits lymphoproliferation while at low concentrations the effect is reversed, causing lymphoproliferation (84). Further, compounds in honey propolis inhibits various viruses such as dengue virus type 2, herpes simplex virus, human cytomegalovirus, influenza virus A1 (85). Together, with immunomodulatory and antiviral effects, propolis can be tried as a prophylactic support against COVID-19.

## Probiotics

The commonly used probiotics are *Bifidobacterium* and *Lactobacillus* species, followed by the *Streptococcus, Enterococcus, Bacillus, and Escherichia coli*. Probiotics not only support the health of the gut but also improves system functioning and regulation (86). Though it is not clear how gut microbiome provides benefit over respiratory tract infections *via* gut-lung axis. In general, it is observed that the gut microbiome impacts systemic immune responses as well as local immune responses at distal mucosal sites, including lungs (87). Consumption of *Bifidobacterium* and *Lactobacillus* have found to help in clearing the influenza virus in the respiratory tract (88). Levels of interferons, mucosal antibodies of lung and activity of NK cells, antigen presenting cells (APCs) are improved by probiotics (89). *Lactobacillus plantarum* DR7 strain has shown to have suppressing effect on the pro-inflammatory cytokines TNF-α, IFN-γ, enhances anti-inflammatory cytokines IL-10, IL-4 and also known to reduce plasma peroxidation levels as well as modulate immune system (90). It is reported that *Lactobacillus acidophilus* CMCC878 administration in mice infected with *Staphylococcus aureus*, and *Pseudomonas aeruginosa* decreased the damage in the lungs by reducing the bacterial load and reducing the inflammation (91). A clinical study has reported that administration of *Leuconostoc mesenteroides* 32-77:1, *Lactobacillus plantarum* 2,362, *L. paracasei ssp. paracasei* 19, *Pediococcus pentosaceus* 5-33:3 along with resistant starch, inulin prebiotics etc. reduced systemic inflammatory response syndrome and other infections (92). *Bifidobacterium longum* BB536 strain prevents infection from influenza and improves innate immunity (93). Though mechanism of their immunomodulating and anti-inflammatory effects in the lung are not clearly understood. In general, probiotics exert anti-inflammatory and immunomodulatory effects *via* modulation of the NF-κB, MAPK and pattern recognition receptors (PRR) pathways that decreases Th2 mediated responses and upregulates Th1 responses. Further, they have an ability to inhibit the attachment of bacterial LPS to CD14 receptor, hence decrease in the overall activation of NF-κB and pro-inflammatory cytokines production (94, 95). Considering the role of probiotics in improving the host innate immune response as well as anti-inflammatory effects (87), and considering the fact that gut involvement and enterocytes (96) can be reservoirs of SARS-CoV-2 infection, probiotics can be repurposed as prophylactics as well as adjuvants to combat the pathogenesis of COVID-19.

## Lactoferrin

Lactoferrin (Lf) is a naturally occurring and non-toxic glycoprotein that has been studied against a broad range of viruses, including SARS-CoV, which is closely related to SARS-CoV-2. Lf inhibits viral entry *via* binding to cell surface molecules or viral particles or both. It was also known to suppress virus replication as in the case of HIV. Therefore, it plays a crucial role in preventing the virus entry and replication (97). Studies have shown that it exerts immunomodulatory and antioxidant effects by inducing the T-cell activation, suppressing the levels of interleukins including IL-6, TNF-α, and downregulating the ferritin (98). Also it suppresses H_2_ O_2_ -induced oxidative stress in human umbilical vein endothelial cells (99). Furthermore, zinc saturated Lf exerts a more potent antiviral effect (100). It is mainly used as a nutritional additive in infant formulas and clinical studies, with doses ranging from 100 mg to 4.5 g a day for various indications without apparent toxicities. and can be tried as a potential preventive and therapeutics against COVID-19 (98).

## Quercetin

Quercetin is a well-known antioxidant with anti-inflammatory and antiviral bioactive. It inhibits TNF-α production in LPS-induced macrophages (101), IL-8 production in lung A549 cells (102), and mRNA levels of TNF-α and IL-1α in glial cells (103). It also limits the production of cyclooxygenase (COX) and lipoxygenase (LOX) enzymes in rat liver epithelial cells (104). Studies have also shown that quercetin has antiviral effects on both RNA and DNA viruses. It inhibits the virus entry and viral-cell fusion (105) and reduces the expression of pro-inflammatory cytokines and lung inflammation induced by rhinovirus in mice (106). Further, quercetin metabolite (4',5-diacetyloxy-3,3',7-trimethoxyflavone) has been shown to inhibit the picornavirus replication by inhibiting the RNA replicase complex (107). Studies have also found that quercetin-3β-galactoside due to the presence of hydroxyl group, it binds to viral protease 3CL_pro_ and inhibits its proteolytic activity (108). In the context of SARS virus infection, supercomputer SUMMIT drug-docking screen and gene set enrichment analyses (GSEA) finds that quercetin, VD, and estradiol interferes the functioning of 85, 70 and 61% of the SARS-CoV-2 viral proteins in human cells, respectively. Based on these observations the study also predicts tripartite combination (quercetin/VD/estradiol) compared to bipartite (VD/quercetin) of may affect 73% human genes encoding SARS-CoV-2 targets implicating a robust mitigating agents against the COVID-19 (109). Further, increased ability of estradiol in affecting human genes encoding SARS-CoV-2 targets compared to testosterone suggests a plausible explanation of the apparently higher male mortality in this corona pandemic (109). In line with these observations, a randomized interventional clinical trial using estradiol or VD as a mitigating agent have been listed on the clinical trial (https://clinicaltrials.gov/ct2/show/NCT04359329).

Furthermore, as observed in prediction models that quercetin binds SARS-CoV-2 S-protein at its host receptor region or to the S-protein-human ACE2 interface interfering the virus entry into cells indicating its therapeutic potential (110). This prediction is consistent with the reports that both quercetin and a structurally similar luteolin inhibits the SARS-CoV virus infection (111). Additionally, other studies have also found that quercetin in combination with VC induces synergistic antiviral and immunomodulatory effects against COVID-19 (47). Taken together, various studies suggest that quercetin possesses potential anti-SARS-CoV-2 effects and can be repurposed as a preventive and therapeutic candidate to combat COVID-19.

## Conclusions

Currently, there is one corona vaccine, Sputnik V, developed by the Gamaleya Research Institute, Moscow has been approved by the Ministry of Health, Russian Federation. It was fast-tracked for use as a corona vaccine, but experts have expressed concern about the vaccine's efficacy and safety since it has not yet been evaluated in phase 3 clinical trials. Presently, there are over 100 vaccines around the world in various stages of research and development. A few of them are in human clinical trials and are being tested rigorously for their safety, efficacy, and dosage standardization. Similarly, there are several drug candidates that have been identified and most are in various stages of research and development, whilst some of them have been repurposed and approved for emergency use in this pandemic. The notable ones approved for use in an emergency include hydroxychloroquine, favipiravir, remdesivir, tocilizumab, etc., Furthermore, no substantial research supports the use of specific food supplements as adjuvant therapy for the management of COVID-19 subjects. The plethora of existing literature provides the scientific evidence on immune-boosting, anti-inflammatory, antioxidant, and antiviral properties of several phytonutrients as summarized in Table 1. Initial studies find that some of these have been found to possess anti-SARS-CoV-2 effects and are being fast-tracked into clinical trials (Table 2). Repurposing of these nutrients in the right combination to achieve the functional synergy in the form of ready-to-eat food supplements may provide both prophylactic and adjuvant therapy against COVID-19.

**Table 1:** Summary of food supplements and their major functional effects.

**Serial number**	**Food supplements**	**Properties**	**Status of the clinical trial**
1	Zinc (Zn) (Antiviral)	◾Protects against oxidative stress and inhibit TNF-α, IFN-γ, FasR and JAK-STAT signaling pathways. ◾Modulates the viral entry, fusion, replication, viral protein translation and virus budding of respiratory viruses.	Phase 1 and 2
2	Vitamin D (VD) (Immune-boosting anti-inflammatory)	◾Blocks NF-κB p65 activation *via* up-regulation of I-kappa-B-alpha (I*K*B-α). ◾Decreases the expression of the pro-inflammatory type 1 cytokines: IL-12, IL-16, IL-8, TNF-α and IFN-γ and increases type 2 cytokines IL-4, IL-5, IL-10. ◾Upregulates the levels of antioxidant NRF-2, facilitates balanced mitochondrial functions.	Phase 2
3	Vitamin C (VC) (Immune-boosting, antioxidant)	◾Decreases pro-inflammatory cytokines, TNF-α and IFN-γ and increases anti-inflammatory IL-10 production. ◾Reduces the duration and severity of upper respiratory infections (viral infections). ◾Scavenges ROS, prevents lipid peroxidation, and protein alkylation and thus protect cells from oxidative stress induced cellular damage.	Phase 2
4	Curcumin (Immune-boosting, antiviral, anti-inflammatory, and antioxidant)	◾Stimulates host interferon production to activate the host innate immunity. ◾Binds to S protein at RBD and ACE2 receptor and inhibits virus entry. ◾Inhibits NF-κB, cyclinD1, COX-2, TNF-α, and STAT signaling pathways. ◾Neutralizes free radicals and enhances the production of antioxidant enzymes.	Phase 1 and 2
5	Cinnamaldehyde (Anti-inflammatory)	◾Suppress the NF-κB, TLR4, and NLRP3 signaling pathways. ◾Downregulates the production of prostaglandins.	Phase 2
6	Allicin (Antiviral, anti-inflammatory)	◾Downregulates the proinflammatory cytokines and inhibits the nitric oxide synthase expression in macrophages. ◾Possess antiviral effect on broad spectrum of viruses of HSV family, parainfluenza virus and human rhinovirus.	Phase 1 and 2
7	Piperine (Anti-inflammatory and antioxidant)	◾Reduces the production of the IL-1β, IL-6, TNF-α, COX-2, nitric oxide synthase-2, and NF-κB. ◾Neutralizes free radicals, ROS, and hydroxyl radicals.	Phase 2
8	Selenium (Immune-boosting, and antioxidant)	◾Promotes the T cell proliferation, NK cell activity and innate cell function. ◾It downregulates the proinflammatory cytokines (IL-1β and IL-6). ◾Augments glutathione peroxidase and other antioxidant selenoenzymes activities.	Phase 2
9	Propolis (Immune-boosting, anti-inflammatory)	◾Exhibits the immunomodulatory effect through extracellular signal-regulated kinase 2 and MAPK pathways and by modulating the NFAT and NF-κB activation. ◾Inhibits various viruses such as dengue virus type 2, herpes simplex virus, human cytomegalovirus, influenza virus A1.	
10	Probiotics (Immune boosting, anti-inflammatory)	◾*Lactobacillus plantarum* DR7 suppress proinflammatory cytokines TNF-α, IFN-γ, enhances anti-inflammatory cytokines IL-10, IL-4. ◾*Lactobacillus acidophilus* CMCC878 reduces the bacterial load and inflammation in mice lungs infected with staphylococcus and pseudomonas. ◾*Leuconostoc mesenteroides* 32-77:1, *L. plantarum* 2,362, *L. paracasei ssp. paracasei* 19, *Pediococcus pentosaceus* 5-33:3 along with resistant starch, inulin etc reduce systemic inflammatory response syndrome and other infections. ◾*Bifidobacterium longum* BB536 prevents infection from influenza and improves innate immunity.	Phase 2
11	Lactoferrin (antiviral)	◾Downregulates the IL-6, TNF-α, and ferritin. ◾Inhibits the viral entry and suppress the viral replication.	Phase 1 and 2
12	Quercetin (antiviral)	◾Inhibits the production of the TNF-α, IL-8, IL-1α, COX, and LOX enzymes. ◾Possesses antiviral effects against both RNA (influenza and coronavirus) and DNA viruses (herpesvirus). ◾Act as ligand for the S protein of virus and ACE 2 and interferes in binding of virus to cells.	Phase 1 and 2

**Table 2:** Registered clinical trials of food supplements (Source: ClinicalTrials.gov).

**Serial number**	**Food supplements**	**Registered clinical trials for COVID-19**
1	Zinc (Zn)	1. Evaluation of the Relationship Between Zinc Vitamin D and b12 Levels in the COVID-19 positive Pregnant Women (Pinar Yalcin Bahat Istanbul, Istanbul, Turkey). 2. Zinc with Chloroquine/Hydroxychloroquine in Treatment of COVID-19 (Tanta University hospital Tanta, Egypt). 3. A Study of Hydroxychloroquine, Vitamin C, Vitamin D, and Zinc for the Prevention of COVID-19 Infection (Progena Biome Ventura, California, United States).
2	Vitamin D (VD)	1. COVID-19 and Vitamin D in Nursing- home (Angers University Hospital Angers, France). 2. Vitamin D Supplementation in Patients with COVID-19 (Clinical Hospital of the School of Medicine, University of São Paulo São Paulo Brazil). 3. Vitamin D Testing and Treatment for COVID 19 (Arizona State University Tempe, Arizona, United States).
3	Vitamin C (VC)	1. Administration of Intravenous Vitamin C in the Novel Coronavirus Infection (COVID-19) and Decreased Oxygenation (Hunter Holmes Mcguire veteran Affairs Medical Center Richmond, Virginia, United States). 2. A Study of Hydroxychloroquine, Vitamin C, Vitamin D, and Zinc for the Prevention of COVID-19 Infection (ProgenaBiome Ventura, California, United States). 3. The Study of Quadruple Therapy Zinc, Quercetin, Bromelain and Vitamin C on the Clinical Outcomes of Patients Infected With COVID-19 (Ministry of Health. First health cluster, Riyadh, Riyadh, Saudi Arabia).
4	Curcumin	1. A phase II, Controlled Clinical Study Designed to Evaluate the Effect of ArtemiC in Patients Diagnosed With COVID-19 (Hillel Yaffe Medica Center Hedera, Haifa, Israel Nazareth Hospital EMMS Nazareth, North, Israel Rambam Health Care Campus Haifa, Israel).
5	Probiotics	1. Effect of Lactobacillus on the Microbiome of Household Contacts Exposed to COVID-19 (Duke University Durham, North Carolina, United States). 2. Oxygen-Ozone as Adjuvant Treatment in Early Control of COVID-19 Progression and Modulation of the gut Microbial Flora (Francesco Pugliese Rome, RM Italy). 3. Evaluation of the Probiotic *Lactobacillus Coryniformis* K8 on COVID-19 Prevention in Healthcare Workers (Raquel Rodrigues Blanque Granada, Spain). 4. Study to Evaluate the Effect of a Probiotic in COVID-19 (Hospital Universitario de Vinalopo Elache, Alicante, Spain, Hospital Universitario de Torrevieja Torreviveja, Alicante, Spain).
6	Quercetin	1. Effect of Quercetin on Prophylaxis and Treatment of COVID-19 (Kanuni Sultan Suleyman Training and Research Hospital Istanbul, Turkey). 2. The Study of Quadruple Therapy Zinc, Quercetin Bromelain and Vitamin C on the Clinical Outcomes of Patients Infected With COVID-19 (Ministry of Health. First Health cluster, Riyadh. Riyadh, Saudi Arabia). 3. Estrogen Patch for COVID-19 Symptoms (Stony Brook University Hospital, Stony Brook, New York. USA).

## Author Contributions

MM, VP, RN, and PJ: drafted the article. PH and PVR: edited the article. All authors: contributed to the article and approved the submitted version.

## Conflict of Interest

The authors declare that the research was conducted in the absence of any commercial or financial relationships that could be construed as a potential conflict of interest.
